# Adjustment of Respiration Strategies in Roots Contributes to the Waterlogging Resistance in *Actinidia valvata* ‘Shuixiu’

**DOI:** 10.3390/ijms27073147

**Published:** 2026-03-30

**Authors:** Lingling Xu, Ping Yuan, Qiaosheng Jiang, Fanjing Zhang, Qing Luo, Shibiao Liu, Yan Wang, Jianyou Gao, Manrong Zha

**Affiliations:** 1 School of Life Sciences, Jishou University, Jishou 416000, China; x18379268826@163.com (L.X.); bbh18230580962226@163.com (F.Z.); 15243949776@163.com (Q.L.); liushibiao_1@163.com (S.L.); wy408@jsu.edu.cn (Y.W.); 2 Hunan Horticulture Research Institute, Hunan Academy of Agricultural Sciences, Changsha 410125, China; yuanping@hunaas.cn; 3 Guangxi Institute of Botany, Guangxi Zhuang Autonomous Region and Chinese Academy of Sciences, Guilin 541006, China; 13878340927@163.com (Q.J.); gaojian19970802@163.com (J.G.)

**Keywords:** waterlogging stress, transcriptome, sugar metabolism, respiratory metabolism, *Actinidia valvata* ‘Shuixiu’

## Abstract

Soil hypoxia caused by waterlogging severely restricts kiwifruit growth, and screening waterlogging-tolerant rootstocks and analyzing their mechanisms are of great significance for industrial development. In this study, waterlogging-tolerant *Actinidia valvata* ‘Shuixiu’ was used as the test material and *Actinidia chinensis* ‘Hongyang’ as the control. Waterlogging stress was simulated artificially, and physiological measurements combined with transcriptome sequencing were used to explore its waterlogging tolerance regulatory characteristics based on respiratory metabolism. The results showed that the waterlogging tolerance of ‘Shuixiu’ was significantly better than that of ‘Hongyang’. It upregulated sucrose synthase and α/β-amylase genes and inhibited the continuous up-regulation of trehalose-6-phosphate synthase genes, leading to significant accumulation of glucose-6-phosphate, a key glycolytic substrate. Some members of glycolytic key gene families, such as glucose-6-phosphate isomerase and phosphofructokinase, were upregulated in ‘Shuixiu’, which increased phosphoglycerate kinase activity and accumulated 3-phosphoglyceric acid and pyruvate, ensuring efficient conversion of carbon sources to ATP. Some members of core tricarboxylic acid cycle gene families, such as pyruvate dehydrogenase and citrate synthase, were upregulated in ‘Shuixiu’, with significantly higher pyruvate dehydrogenase activity and acetyl coenzyme A content, maintaining partial aerobic respiration capacity. Some members of the alanine transaminase gene family were upregulated in ‘Shuixiu’ to enhance alanine fermentation, resulting in a significant reduction in root ethanol accumulation. This study clarified the core respiratory metabolic regulatory characteristics of kiwifruit in response to waterlogging and provided key targets and a theoretical basis for molecular breeding of waterlogging-tolerant rootstocks.

## 1. Introduction

Waterlogging severely impacts crop productivity, with yield reductions reaching up to 80% in affected areas such as the middle-lower Yangtze River basin in China, where poor drainage is common [1,2,3]. Soil hypoxia caused by waterlogging triggers a rapid shift in root metabolism from aerobic respiration to anaerobic respiration. This inefficient pathway depletes large quantities of energy reserves in plants and leads to the accumulation of toxic substances, such as lactate and ethanol. Meanwhile, hypoxia inhibits the normal function of the electron transport chain, resulting in the accumulation of electrons and reducing power, which in turn promotes the excessive production of reactive oxygen species (ROS) [4,5]. The consequent imbalance in ROS metabolism can readily induce lipid peroxidation and damage the structure of cell membranes, proteins, and lipids. Collectively, these physiological disruptions—including energy deficit, accumulation of toxic substances, and oxidative stress—compromise the systemic functions of both roots and shoots, ultimately inhibiting plant growth [6].

Kiwifruit (*Actinidia* spp.), a deciduous woody vine of the Actinidiaceae family, possesses a shallow, fleshy root system that is highly sensitive to water conditions in most species. While preferring a moist environment, kiwifruit is intolerant of waterlogging and is considered one of the most waterlogging-sensitive fruit tree species [7]. According to the Sichuan Academy of Agricultural Sciences, persistent heavy rainfall in 2018 damaged a total of 2866.67 hectares of kiwifruit orchards across Sichuan Province. In 2020, continuous torrential rain affected approximately 800.00 hectares of kiwifruit plantations in Mianzhu City, of which about 266.67 hectares were damaged and nearly 133.33 hectares suffered severe damage. Currently, kiwifruit is predominantly propagated by grafting, making the waterlogging tolerance of the rootstock crucial for the overall resilience of the plant [8]. Therefore, selecting and breeding waterlogging-tolerant rootstocks and elucidating their resistance mechanisms are of great significance for mitigating losses in the kiwifruit industry caused by flooding. In recent years, some orchards in China have begun to use rootstocks locally known as ‘Shuiyangtao’. However, ‘Shuiyangtao’ was not a single taxon but a common local name applied to a group of kiwifruit species, including *A. valvata*, *A. macrosperma*, *A. melanandra*, and *A. lanceolata*. As these germplasms have not undergone systematic selection, their effects on the growth and development of the scion are inconsistent. Consequently, researchers have focused on screening for waterlogging-tolerant germplasms from these genetic resources. For instance, Geng et al. [9] identified two promising waterlogging-tolerant accessions, ‘0904’ and ‘Jinong No. 1’, from 41 *A. arguta* resources, while Xu et al. [10] reported the strong waterlogging resistance of the *A. valvata* accession DJY-C-1. The *A. valvata* ‘Shuixiu’, recently selected by our team, is a rootstock germplasm with outstanding potential for waterlogging tolerance. Nevertheless, the physiological and molecular mechanisms underlying its waterlogging resistance remain unclear.

Regarding morphological adaptations, studies have shown that the waterlogging-tolerant genotype *A. valvata* KR5 developed adventitious roots at the stem base near the water surface following waterlogging, thereby enhancing oxygen uptake [11,12]. These adventitious roots typically possessed well-developed aerenchyma, which effectively replaced the original root system impaired by hypoxia, improving the efficiency of water and nutrient absorption and alleviating water stress in the aerial parts [13,14]. Furthermore, KR5 formed hypertrophic lenticels on the root collar [15]. These hypertrophic lenticels served as crucial pathways for oxygen entry into plants. Their enlarged intercellular spaces connected directly with the secondary aerenchyma in the stem, forming a low-resistance gas diffusion pathway. Driven by the oxygen partial pressure gradient, oxygen was efficiently transported to submerged roots via passive diffusion, thereby improving root oxygen supply and alleviating hypoxia stress caused by waterlogging. Furthermore, the formation of hypertrophic lenticels facilitated the release of carbon dioxide produced by plant respiration, maintaining the balance of internal gas exchange [16,17]. Tolerant kiwifruit root systems form aerenchyma in the root cortex through ethylene- and reactive oxygen species (ROS)-mediated programmed cell death. Although the increased ethylene levels facilitated aerenchyma formation, they could also induce morphological changes such as swelling of the root-shoot junction, epinastic growth of petioles, and inhibition of shoot growth, consequently restraining plant development to some extent [14,18]. *A. valvata* also enhanced its adaptability under waterlogging stress by increasing proline content [19]. Additionally, it was found that after 32 days of waterlogging, the genotype ‘Zhemizhen-1’ significantly upregulated the activities of superoxide dismutase (SOD) and peroxidase (POD), effectively scavenging ROS accumulated due to hypoxia and preventing membrane lipid peroxidation damage [20].

Hypoxia stress caused by waterlogging is the main factor leading to root damage in kiwifruit. In response, kiwifruit plants developed a series of adaptive mechanisms related to respiratory metabolism. For example, under waterlogging conditions, overexpressing the transcription factor *AvERF078* in *Actinidia chinensis* cv. ‘Hongyang’ resulted in greater accumulation of soluble sugars in roots, which provided sufficient substrates for respiration. This might represent an important metabolic basis for enhanced waterlogging tolerance regulated by *AvERF078* [15]. In addition, overexpression of the transcription factor AvERF73 also significantly improved hypoxia tolerance in kiwifruit cultivar ‘Hongyang’, although its detailed regulatory mechanism remains to be further investigated [21]. Alcohol dehydrogenase (ADH) and pyruvate decarboxylase (PDC) were key enzymes in the ethanol fermentation pathway. Studies showed that roots of *Actinidia deliciosa*, which exhibited stronger hypoxia tolerance, displayed significantly higher ADH activity than those of hypoxia-sensitive *A*. *chinensis*. This suggested that under hypoxia stress, *A. deliciosa* more easily maintained a higher rate of ethanol fermentation in roots, which favored the regeneration of NAD^+^ and thus ensured continuous energy supply (ATP production) via glycolysis [22]. However, it should be noted that the accumulation of ethanol and acetaldehyde is one of the major causes of cellular injury under rhizosphere hypoxia [23]. In the waterlogging-tolerant kiwifruit genotype *A. valvata* Dunn, the expression of ADH and PDC genes was upregulated by waterlogging stress but only moderately induced during the early stage (first 2 days), followed by a significant decrease. In contrast, these two genes remained highly expressed in the hypoxia-sensitive cultivar *A. chinensis* cv. ‘Donghong’. Furthermore, the transcription factor *AcMYB68* directly activated the expression of *AcPDC2*, while *AcERF74/75* positively regulated *ADH* expression. However, the transcript levels of *AcMYB68* and *AcERF74/75* were both suppressed in the waterlogging-tolerant *A. valvata* Dunn [24]. Taken together, distinct kiwifruit species or cultivars exhibit divergent adaptive pathways of respiratory metabolism under hypoxia stress, which determine their differential waterlogging tolerance.

There existed extensive variation in waterlogging tolerance among kiwifruit cultivars. This study selected the waterlogging-tolerant cultivar ‘Shuixiu’ (previously identified by our team via systematic screening), but its physiological and molecular regulatory mechanisms of respiratory metabolism under waterlogging stress remained unclear. Combining physiological measurements and comparative transcriptomic analysis, we systematically explored the respiratory metabolism regulation of ‘Shuixiu’ under waterlogging to clarify key regulatory pathways. Although many studies had focused on kiwifruit waterlogging tolerance, no systematic analysis had targeted the specific cultivar ‘Shuixiu’. This study filled this gap, enriched the relevant theoretical framework, and provided theoretical support for molecular breeding of waterlogging-tolerant rootstocks and the sustainable development of the kiwifruit industry.

## 2. Results

### 2.1. Compared with ‘Shuixiu’, ‘Hongyang’ Exhibits Superior Tolerance to Waterlogging Stress

**Figure 1:**
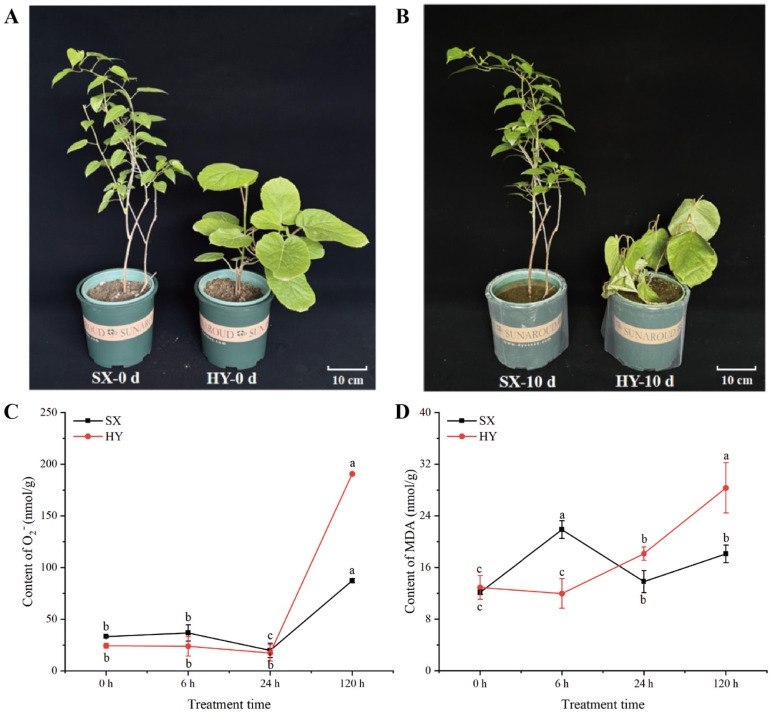
Phenotypic effects of waterlogging stress on ‘Shuxiu’ and ‘Hongyang’. (**A**) Plant growth of ‘Shuxiu’ and ‘Hongyang’ before waterlogging. (**B**) Plant growth status of ‘Shuxiu’ and ‘Hongyang’ after 10 days of waterlogging. (**C**) Changes in superoxide anion levels in the roots of ‘Shuxiu’ and ‘Hongyang’ after waterlogging. (**D**) Changes in MDA content in the roots of ‘Shuxiu’ and ‘Hongyang’ after waterlogging; SX: ‘Shuixiu’, HY: ‘Hongyang’. Lowercase letters denote statistically significant differences between periods within the same group (*p* < 0.05).

After waterlogging treatment, plants of ‘Shuixiu’ and ‘Hongyang’ showed distinct phenotypic differences. Following 10 days of waterlogging, ‘Shuixiu’ leaves exhibited only slight wilting and chlorosis (Figure 1A), whereas ‘Hongyang’ plants displayed severe wilting symptoms (Figure 1B). To further explore the underlying physiological mechanisms, reactive oxygen species levels and membrane lipid peroxidation in roots were determined. For reactive oxygen metabolism, the content of superoxide anion in ‘Hongyang’ roots increased to 190.71 nmol/g after 120 h of waterlogging, while that in ‘Shuixiu’ was 87.32 nmol/g, with a significant difference between the two genotypes (*p* < 0.05) (Figure 1C). For membrane lipid peroxidation, the malondialdehyde (MDA) content in ‘Shuixiu’ roots rose to 21.86 nmol/g at 6 h after waterlogging, representing an increase of 80.19% relative to the initial level, and then decreased slightly to 18.10 nmol/g at 120 h. In contrast, MDA content in ‘Hongyang’ roots increased continuously after waterlogging and reached 28.33 nmol/g at 120 h, which was significantly higher than that in ‘Shuixiu’ at the same time point (Figure 1D, *p* < 0.05). Comprehensive phenotypic and physiological analysis showed that after waterlogging treatment, ‘Hongyang’ not only suffered more severe phenotypic damage, but its root superoxide anion accumulation and MDA content were also significantly higher than those of ‘Shuixiu’, indicating that ‘Hongyang’ exhibited a more obvious phenotypic response to waterlogging stress and suffered more severe oxidative damage than ‘Shuixiu’.

### 2.2. Transcriptome Analysis of ‘Shuixiu’ and ‘Hongyang’ in Response to Waterlogging

**Figure 2:**
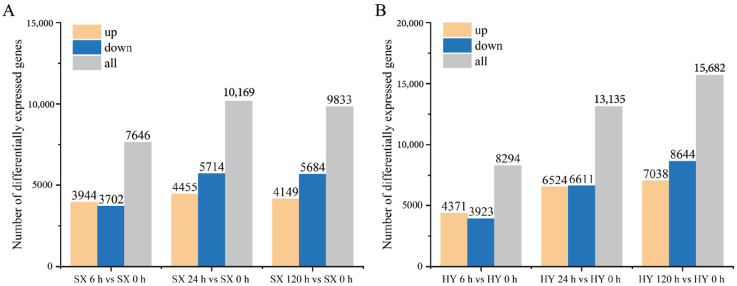
Identification of differentially expressed genes. (**A**) ‘Shuixiu’. (**B**) ‘Hongyang’; SX: ‘Shuixiu’, HY: ‘Hongyang’.

To investigate the adaptation mechanisms of ‘Shuixiu’ and ‘Hongyang’ to waterlogging stress, high-throughput RNA-Seq analysis was performed on 24 cDNA libraries constructed before and after waterlogging treatment. The number of clean reads ranged from 19,272,205 to 28,434,460, the Q30 value of each library was higher than 93.42%, and the average GC content was 45.68% (Table S1). Gene annotation was carried out using the NR, NT, Pfam, KOG/COG, Swiss-Prot, KO, and GO databases, and a total of 48,081 genes were identified (Table S2). Using SX 0 h and HY 0 h as controls, 24,339 differentially expressed genes (DEGs) were screened with the thresholds of |log_2_FC| ≥ 1.0 and padj < 0.05. The distribution of DEGs at each treatment time point is shown in Figure 2. In ‘Shuixiu’, 7646 DEGs (3499 upregulated and 3702 downregulated) were identified at 6 h of waterlogging; 10,169 DEGs (4455 upregulated and 5714 downregulated) at 24 h; and 9833 DEGs (4119 upregulated and 5684 downregulated) at 120 h (Figure 2A). In ‘Hongyang’, 8294 DEGs (4371 upregulated and 3923 downregulated) were detected at 6 h of waterlogging; 13,135 DEGs (6524 upregulated and 6611 downregulated) at 24 h; and 15,682 DEGs (7038 upregulated and 8684 downregulated) at 120 h (Figure 2B). Comparative analysis revealed that the number of DEGs in ‘Hongyang’ was higher than that in ‘Shuixiu’ at all time points during the entire waterlogging treatment, indicating that ‘Hongyang’ exhibited a stronger molecular response to waterlogging treatment.

### 2.3. Analysis of Differentially Expressed Genes Related to Sugar Conversion and Physiological Indicators

**Figure 3:**
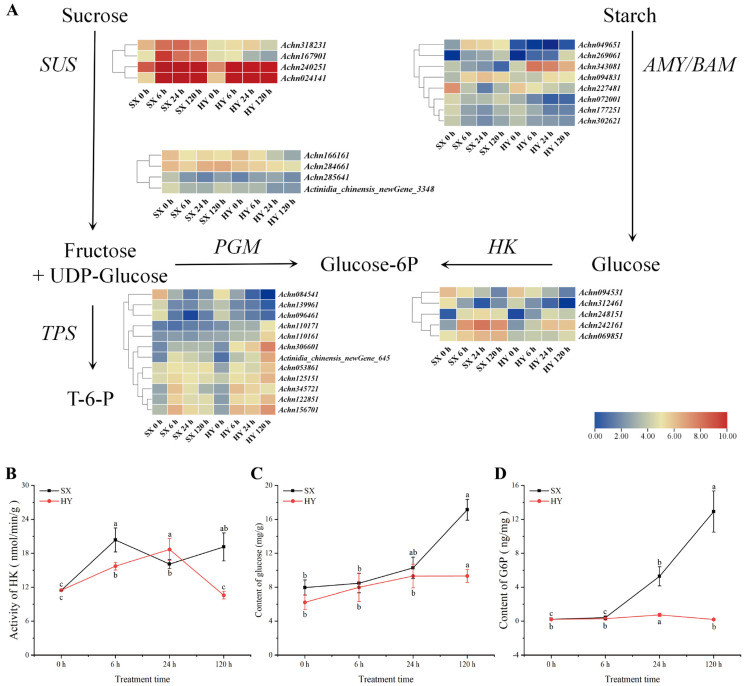
Integration of transcriptome and physiological data of the starch and sucrose metabolic pathway. (**A**) Differential expression analysis of starch and sucrose metabolic pathways, SUS: sucrose synthase; PGM: phosphoglucomutase; TPS: trehalose-phosphate synthase; HK: hexokinase; AMY/BAM: α-amylase/β-amylase. (**B**) Changes in HK enzyme activity. (**C**) Changes in glucose content. (**D**) Changes in glucose-6-phosphate content; SX: ‘Shuixiu’, HY: ‘Hongyang’; Lowercase letters denote statistically significant differences between periods within the same group (*p* < 0.05).

Through transcriptome analysis, 33 DEGs involved in sugar conversion were identified, including five hexokinase (HK) genes, four sucrose synthase (SUS) genes, eight α-/β-amylase (AMY/BAM) genes, four phosphoglucomutase (PGM) genes, and 12 trehalose-6-phosphate synthase (TPS) genes (Figure 3A). The gene expression profiles exhibited cultivar-specific patterns between ‘Shuixiu’ and ‘Hongyang’. Specifically, *Achn069851* (HK) was upregulated only in ‘Shuixiu’; *Achn167901* (SUS) and *Achn049651* (BAM) were upregulated in ‘Shuixiu’ but downregulated in ‘Hongyang’, whereas *Achn343081* (BAM) showed the opposite expression pattern. Among the TPS genes, three were downregulated in both ‘Shuixiu’ and ‘Hongyang’, while five genes (*Achn125151*, *Achn122851*, *Achn345721*, *Achn156701*, and *Actinidia_chinensis_newGene_645*) were upregulated in both cultivars. Moreover, these five TPS genes reached their peak expression at 6 h of waterlogging and then decreased in ‘Shuixiu’, whereas they continued to be upregulated in ‘Hongyang’.

Given that HK (hexokinase) and PGM (phosphoglucomutase) genes exhibited cultivar-specific transcriptional differences and that these genes directly regulate glucose phosphorylation and the metabolic flux of glucose-6-phosphate (G6P), respectively, the HK activity, glucose content, and G6P content in the roots of both cultivars were measured to further verify the physiological manifestations of these transcriptional changes. After waterlogging, the change patterns of root HK activity in ‘Shuixiu’ and ‘Hongyang’were obviously different. In ‘Hongyang’, HK activity peaked at 24 h (18.67 nmol/min/g) and then declined sharply, falling below the pre-waterlogging level (11.48 nmol/min/g) by 120 h (10.59 nmol/min/g). In contrast, HK activity in ‘Shuixiu’ remained above the pre-waterlogging level throughout the experimental period and was significantly higher than that in ‘Hongyang’ at 6 h and 120 h (Figure 3B). Glucose content changes were correlated with HK activity. Following waterlogging, glucose content in the roots of both cultivars showed an increasing trend, and at 120 h, the glucose content in ‘Shuixiu’ (17.13 mg/g) was 1.84-fold higher than that in ‘Hongyang’ (9.33 mg/g) (Figure 3C). As a key intermediate in carbohydrate conversion and respiratory metabolism, the difference in G6P content between the two cultivars was even more pronounced. G6P content in ‘Shuixiu’ roots increased continuously and significantly after waterlogging, whereas in ‘Hongyang’ roots, it only increased transiently during the first 24 h and then gradually decreased to a relatively low level. The difference in G6P content between the two cultivars was highly significant at 24 h (*p* < 0.05), and this difference continued to widen with prolonged waterlogging. At 120 h, the G6P content in ‘Shuixiu’ was 64.45-fold higher than that in ‘Hongyang’ (Figure 3D). These results further confirmed that under waterlogging stress, the ‘Shuixiu’ accumulated higher levels of glucose-6-phosphate (G6P) by regulating sugar conversion processes and thereby provided sufficient substrates for subsequent respiratory metabolism.

### 2.4. Analysis of Glycolysis-Related Gene Expression and Physiological Indicators

**Figure 4:**
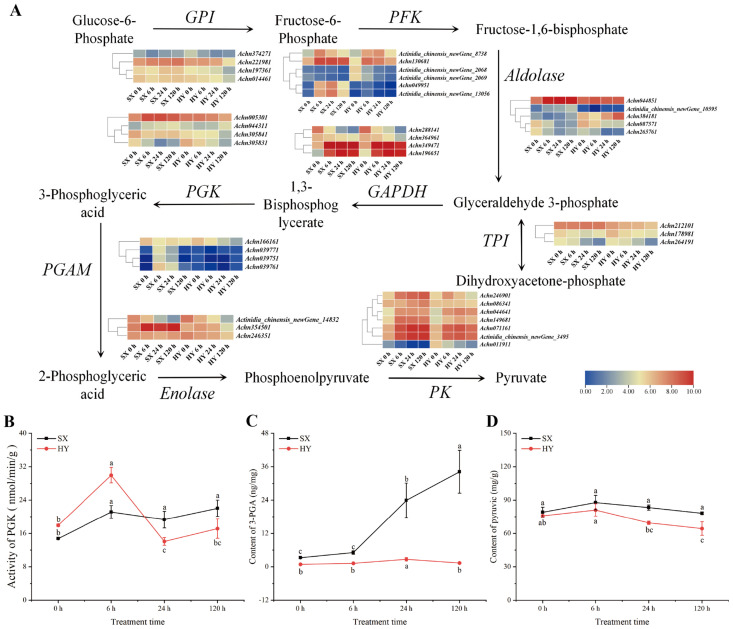
Integration of transcriptome and physiological data of the glycolytic pathway. (**A**) Differential expression analysis of the glycolytic pathway. GPI: glucose-phosphate isomerase; PFK: phosphofructokinase; GAPDH: glyceraldehyde 3-phosphate dehydrogenase; PGK: phosphoglycerate kinase; PGAM: phosphoglyceromutase; PK: pyruvate kinase. (**B**) Changes in PGK enzyme activity. (**C**) Changes in 3-PGA content. (**D**) Changes in pyruvate content; SX: ‘Shuixiu’, HY: ‘Hongyang’; Lowercase letters denote statistically significant differences between periods within the same group (*p* < 0.05).

Glycolysis was a key process for energy production via plant respiration, and its activity directly affected root energy supply. A total of 40 glycolysis-related genes were identified as differentially expressed under stress via transcriptome analysis (Figure 4C). Among them, four genes encoding glucose-6-phosphate isomerase (GPI) were all significantly downregulated in ‘Hongyang’, whereas in ‘Shuixiu’, only *Achn221981* was specifically upregulated, and the other GPI genes showed no significant differential expression. Of the six differentially expressed phosphofructokinase (PFK) genes, *Achn049951* and *Actinidia_chinensis_newGene_13056* were specifically activated and upregulated in ‘Shuixiu’. In contrast, *Actinidia_chinensis_newGene_2068* and *Actinidia_chinensis_newGene_2069* remained stably expressed in ‘Shuixiu’ but were significantly downregulated in ‘Hongyang’. Furthermore, multiple genes encoding enzymes involved in the ATP-generating phase of glycolysis were specifically upregulated in ‘Shuixiu’, including two aldolase genes (*Achn044851*, *Actinidia_chinensis_newGene_10595*), two phosphoglycerate kinase (PGK) genes (*Achn005301*, *Achn044311*), three phosphoglycerate mutase (PGAM) genes (*Achn039751*, *Achn039761*, *Achn039771*), the enolase gene *Achn354501*, and the pyruvate kinase gene *Achn086341*. In comparison, the pyruvate kinase (PK) gene *Achn011911* was significantly downregulated only in ‘Hongyang’.

We further determined the changes in root phosphoglycerate kinase (PGK) activity, 3-phosphoglycerate (3-PGA) content, and pyruvate content before and after waterlogging. The results showed that PGK activity was higher in ‘Hongyang’ than in ‘Shuixiu’ before waterlogging and at 6 h of waterlogging. Subsequently, PGK activity decreased in ‘Hongyang’ but increased in ‘Shuixiu’. At 24 h, PGK activity in ‘Shuixiu’ was significantly higher than that in ‘Hongyang’ (Figure 4B, *p* < 0.05). Measurements of 3-PGA content revealed that 3-PGA content was higher in ‘Shuixiu’ than in ‘Hongyang’ both before and during waterlogging, and the difference between the two cultivars gradually widened with increasing waterlogging duration. At 120 h, the 3-PGA content was 34.18 ng/mg in ‘Shuixiu’ but only 1.35 ng/mg in ‘Hongyang’ (Figure 4C, *p* < 0.05). For pyruvate content, no significant difference was observed between the two cultivars before waterlogging. After waterlogging, root pyruvate content showed a decreasing trend in ‘Hongyang’, whereas it remained relatively stable in ‘Shuixiu’ throughout the treatment and was higher than in ‘Hongyang’ at all post-waterlogging time points (Figure 4D). These results indicated that, under waterlogging stress, ‘Shuixiu’ performed better than ‘Hongyang’ in the expression of key glycolytic genes, the maintenance of PGK enzyme activity, and the accumulation of downstream metabolites.

### 2.5. Analysis of Tricarboxylic Acid Cycle-Related Gene Expression and Physiological Indicators

**Figure 5:**
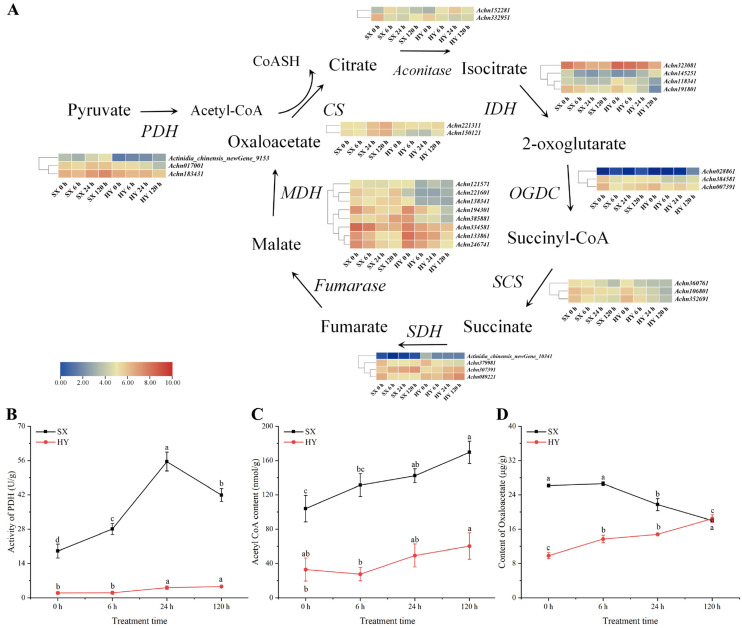
Integration of transcriptome and physiological data of the tricarboxylic acid (TCA) cycle. (**A**) Differential expression analysis of the tricarboxylic acid (TCA) cycle, PDH: pyruvate dehydrogenase; CS: citrate Synthase; IDH: isocitrate dehydrogenase; OGDC: 2-oxoglutarate dehydrogenase complex; SCS: succinyl-CoA synthetase; SDH: succinate dehydrogenase; MDH: malate dehydrogenase. (**B**) Changes in PDH enzyme activity. (**C**) Changes in acetyl-CoA content. (**D**) Changes in oxaloacetate content; SX: ‘Shuixiu’, HY: ‘Hongyang’. Lowercase letters denote statistically significant differences between periods within the same group (*p* < 0.05).

The tricarboxylic acid (TCA) cycle, as the core mechanism of aerobic respiration, is a key pathway for plants to efficiently obtain energy through the metabolism of carbohydrates and other molecules. In this study, 29 TCA cycle-related genes showed significant differential expression, among which most genes exhibited a downregulated expression trend in both ‘Hongyang’ and ‘Shuixiu’ (Figure 5A). This indicates that waterlogging stress leads to hypoxia in kiwifruit roots, thereby inhibiting aerobic respiration. Furthermore, by comparing the differential expression trends of genes between ‘Hongyang’ and ‘Shuixiu’, we found that several key genes displayed cultivar-specific characteristics: among the three genes encoding PDH, *Achn017001* and *Achn183431* were upregulated in ‘Shuixiu’ but downregulated in ‘Hongyang’; the genes *Achn221311* and *Achn150121*, which encode citrate synthase, were also only upregulated in SX; among the seven differentially expressed genes encoding malate dehydrogenase (MDH), *Achn138341* and *Achn385881* also showed the cultivar-specific feature of ‘upregulation in ‘Shuixiu’ and downregulation in ‘Hongyang’’. Based on the above-mentioned differential gene expression, we hypothesized that SX might maintain higher aerobic respiration efficiency under waterlogging stress.

We further determined the changes in root pyruvate dehydrogenase (PDH) activity and the contents of acetyl-CoA and oxaloacetate before and after waterlogging. The assay of PDH activity showed that PDH activity was significantly higher in ‘Shuixiu’ (19.04 U/g) than in ‘Hongyang’ (1.96 U/g) before waterlogging (*p* < 0.05). After waterlogging, root PDH activity increased in both cultivars, but the increase was much more pronounced in ‘Shuixiu’. At 24 h, PDH activity in ‘Shuixiu’ reached 55.55 U/g, representing a 292% increase compared with the pre-waterlogging level, and was 12.59-fold higher than that in ‘Hongyang’ at the same time point. At 120 h of waterlogging, PDH activity in ‘Shuixiu’ remained at 41.92 U/g, whereas that in ‘Hongyang’ was only 4.61 U/g (Figure 5B). Changes in acetyl-CoA content were consistent with those in PDH activity. Acetyl-CoA content was significantly higher in ‘Shuixiu’ (103.99 nmol/g) than in ‘Hongyang’ (32.92 nmol/g) before waterlogging (*p* < 0.01). After waterlogging, acetyl-CoA content increased in both cultivars, with a more marked increase in ‘Shuixiu’. At 120 h, acetyl-CoA content reached 169.58 nmol/g in ‘Shuixiu’, but only 60.25 nmol/g in ‘Hongyang’ at the same time point (Figure 5C). In contrast to the above indices, oxaloacetate content exhibited an opposite trend. Oxaloacetate content was significantly higher in ‘Shuixiu’ (26.17 μg/g) than in ‘Hongyang’ (9.83 μg/g) before waterlogging (*p* < 0.05). However, after waterlogging, oxaloacetate content decreased significantly in ‘Shuixiu’ but increased significantly in ‘Hongyang’, and the contents in the two cultivars were similar at 120 h (Figure 5D, *p* > 0.05). Taken together, significant differences existed between the two cultivars in the expression patterns of key genes involved in the tricarboxylic acid cycle, the dynamics of PDH activity, and the accumulation of metabolites under waterlogging stress.

### 2.6. Analysis of Genes and Physiological Indices Related to Fermentation Pathways Under Anaerobic Respiration

**Figure 6:**
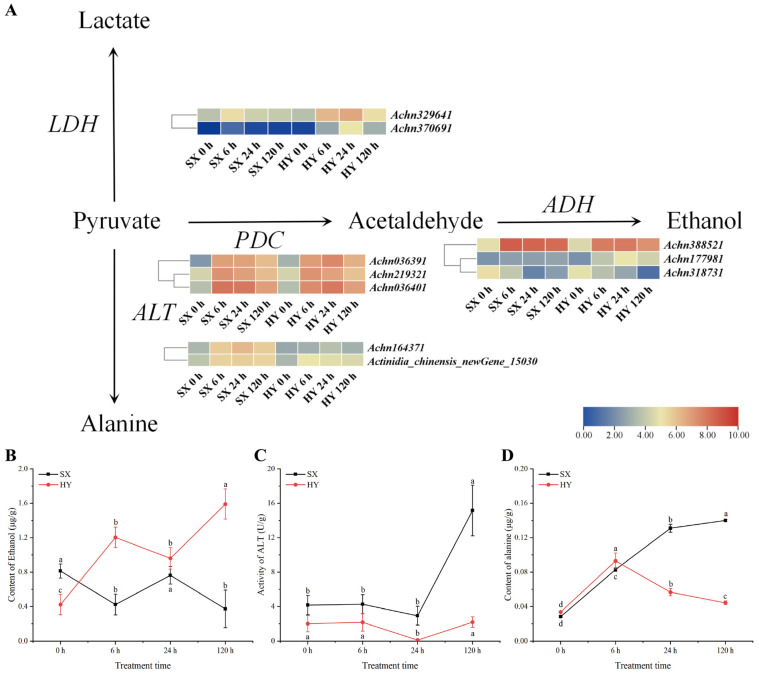
Integrated analysis of transcriptome and physiology from anaerobic respiratory pathways. (**A**) Differential expression analysis of the anaerobic respiratory pathways, PDC: pyruvate decarboxylase; ADH: alcohol dehydrogenase; ALT: alanine transaminase; LDH: lactate dehydrogenase. (**B**) Changes in PDH enzyme activity. (**C**) Changes in acetyl-CoA content. (**D**) Changes in oxaloacetate content; SX: ‘Shuixiu’, HY: ‘Hongyang’. Lowercase letters denote statistically significant differences between periods within the same group (*p* < 0.05).

Under anaerobic conditions, pyruvate produced by glycolysis was mainly metabolized through three fermentation pathways: ethanol fermentation (catalyzed by pyruvate decarboxylase PDC and alcohol dehydrogenase ADH), lactate fermentation (catalyzed by lactate dehydrogenase LDH), and alanine fermentation (catalyzed by alanine transaminase ALT). Transcriptome analysis identified 10 DEGs encoding key enzymes involved in these pathways under waterlogging stress, and these genes showed distinct expression patterns between the two cultivars (Figure 6A). In ‘Hongyang’, the LDH gene *Achn370691* and the ADH gene *Achn177981* were significantly upregulated, whereas this inductive expression pattern was not observed in ‘Shuixiu’. In contrast, the ALT gene *Achn164371,* involved in the alanine pathway, was specifically upregulated in ‘Shuixiu’, while its expression remained largely unchanged in ‘Hongyang’.

To further verify the physiological consequences of the above differential gene expression, we determined the dynamic changes in root ethanol content, ALT enzyme activity, and alanine content in the two cultivars under waterlogging stress. Ethanol content showed opposite trends in the two cultivars: ethanol accumulated rapidly in roots of ‘Hongyang’, reaching 1.59 μmol/g at 120 h, representing a 278.57% increase compared with the pre-waterlogging level. In contrast, ethanol content in ‘Shuixiu’ decreased gradually to 0.37 μmol/g at 120 h, a reduction of 54.32% relative to the pre-waterlogging level, accounting for only 23.27% of that in ‘Hongyang’ at the same time point (Figure 6B). For ALT enzyme activity, the basal ALT activity was higher in ‘Shuixiu’ (4.17 U/g) than in ‘Hongyang’ (2.01 U/g) before waterlogging (*p* < 0.05). After waterlogging, ALT activity in ‘Shuixiu’ exhibited a ‘delayed activation’ pattern: it remained stable during the first 24 h and then increased sharply, peaking at 15.15 U/g at 120 h. In contrast, ALT activity in ‘Hongyang’ remained low throughout the treatment, at only 2.19 U/g at 120 h, representing 14.46% of that in ‘Shuixiu’ at the same time point (Figure 6C). The basal alanine levels were similar in the two cultivars before waterlogging (0.028 μg/g in ‘Shuixiu’ and 0.033 μg/g in ‘Hongyang’), and both increased slightly within 6 h of waterlogging. Subsequently, the trends diverged: alanine content in ‘Shuixiu’ continued to increase to 0.14 μg/g at 120 h, whereas that in ‘Hongyang’ decreased to 0.044 μg/g, accounting for only 31.4% of that in ‘Shuixiu’ at the same time point (Figure 6D). These results indicated that under waterlogging stress, the tolerant cultivar ‘Shuixiu’ redirected pyruvate metabolism toward the alanine fermentation pathway by specifically activating ALT gene expression and enzyme activity, thereby inhibiting the accumulation of ethanol and lactate.

## 3. Discussion

Flooding-induced root hypoxia significantly suppresses aerobic respiration and energy production in kiwifruit, making it a key limiting factor for growth and development [21]. To cope with hypoxic stress, a widely reported adaptive strategy in flooding-tolerant plants is the remodeling of carbohydrate metabolism. This strategy typically accelerated carbohydrate degradation to fuel glycolysis for basal ATP production while maintaining metabolic flux through subsequent fermentation pathways to mitigate hypoxic damage. For example, under low-oxygen conditions, rice synergistically activated AMY/BAM to efficiently hydrolyze starch into soluble sugars, supplying substrates for glycolysis [25,26]. Similarly, the waterlogging-tolerant mung bean genotype T44 significantly upregulates sucrose synthase activity during the early stage of waterlogging, promoting sucrose degradation and enhancing anaerobic energy metabolism [27]. Zhang et al. conducted a transcriptomic analysis of the kiwifruit cultivar ‘Jinkui’ (*Actinidia deliciosa*) before and after waterlogging stress. The results showed that differentially expressed genes were primarily enriched in pathways related to ribosomal function, plant hormone signal transduction, and starch–sucrose metabolism, suggesting that starch and sucrose metabolism may play a regulatory role in the response of this cultivar to waterlogging stress [28]. Additionally, overexpression of the *AcERF74* gene in ‘Hongyang’ was found to significantly promote the accumulation of soluble sugars in roots, thereby enhancing waterlogging tolerance [15]. Taken together, these findings suggest that under waterlogging stress, kiwifruit may increase soluble sugar content in roots to provide energy substrates for respiration under waterlogged conditions, thereby improving tolerance to hypoxic environments. In this study, we further verified and expanded this mechanism in kiwifruit: under waterlogging stress, the waterlogging-tolerant cultivar ‘Shuixiu’ upregulated the expression of several genes encoding sucrose synthase, α-amylase, and β-amylase, thereby accelerating the degradation of sucrose and starch. Hexoses derived from sucrose decomposition and glucose released from starch breakdown were phosphorylated by hexokinase. These two pathways jointly enhanced the supply for glucose-6-phosphate (G6P) synthesis, which was consistent with the sustained accumulation of G6P observed in this study.

G6P occupied a central metabolic hub, serving as a precursor not only for glycolysis but also for the pentose phosphate pathway (PPP), trehalose-6-phosphate synthesis, and starch biosynthesis [29,30,31]. Therefore, the sustained accumulation of G6P in ‘Shuixiu’ raised the question of how this metabolic flux was directed specifically toward glycolysis under waterlogging stress. Our transcriptomic data provided several lines of evidence that glycolysis serves as the predominant sink. First, the expression of G6PDH genes (glucose-6-phosphate 1-dehydrogenase), which encode the rate-limiting enzyme committing G6P to the PPP, was significantly suppressed in both cultivars under waterlogging stress (Table S2). This suppression likely minimized carbon diversion away from glycolysis. Second, key TPS genes, which direct G6P toward trehalose biosynthesis, were actively prevented from sustained upregulation in ‘Shuixiu’ under waterlogging stress, further reducing competition for glycolytic substrate. Third, *phosphoglucomutase* (PGM), which catalyzes the reversible conversion between G6P and glucose-1-phosphate (G1P) for starch biosynthesis, was upregulated in both cultivars (Table S2). However, given the reversible nature of this reaction, the net direction of carbon flux through PGM under hypoxic conditions remains to be determined. Moreover, several key glycolytic genes were significantly induced in ‘Shuixiu’ under waterlogging stress, such as PFK genes and GPI genes, further supporting the active engagement of the glycolytic pathway. Taken together, these findings suggest that the G6P accumulated in ‘Shuixiu’ was likely preferentially channeled into glycolysis under waterlogging stress, which may have contributed to sustaining respiratory metabolism and alleviating energy deficit. This metabolic configuration may therefore represent an important factor underlying the waterlogging tolerance of ‘Shuixiu’.

Trehalose, a non-reducing disaccharide known for its stress-protective functions, can mitigate oxidative damage by upregulating the activities of antioxidant enzymes such as superoxide dismutase (SOD) and ascorbate peroxidase (APX), thereby scavenging excess reactive oxygen species (ROS) [32,33]. Studies indicated that flooding promoted trehalose metabolism in *A. valvata* ‘KR5’, which was associated with the improved submergence tolerance of this cultivar [34]. Our experiment showed that 12 key rate-limiting TPS genes exhibited differential expression patterns in the ‘Shuixiu’ and ‘Hongyang’ after waterlogging treatment, suggesting that trehalose metabolism may be involved in the adaptation of these cultivars to waterlogging stress. However, substantial trehalose synthesis under waterlogging conditions entails a fundamental metabolic contradiction: on one hand, it consumes considerable ATP, and on the other, it competes with glycolysis for limited glucose resources [35]; additionally, high concentrations of trehalose may inhibit starch degradation, restricting efficient carbon utilization [36]. Thus, while trehalose accumulation confers antioxidant protection, it simultaneously imposes costs on energy and carbon metabolism—a conflict that must be resolved for sustainable stress adaptation. In the present study, it was observed that ‘Shuixiu’ actively suppressed the sustained upregulation of key TPS genes under waterlogging stress. This regulatory strategy appeared to represent a precise metabolic trade-off: it limited trehalose overproduction, thereby avoiding ATP depletion and carbon source diversion, while reinforcing the channeling of G6P toward glycolysis as discussed above. Moreover, it prevented the potential inhibitory effects of high trehalose concentrations on starch degradation. By fine-tuning TPS expression, ‘Shuixiu’ effectively resolved the inherent contradiction between trehalose-mediated antioxidant protection and efficient energy/carbon utilization. This regulatory feature likely serves as an important metabolic basis for its strong submergence tolerance.

Glycolysis serves as the central pathway of plant energy metabolism, providing essential ATP and pyruvate through the breakdown of glucose. It plays a crucial role in plant growth, development, and stress responses. Flooding reduces oxygen supply to the roots, prompting plants to initiate anaerobic respiration to cope with hypoxic stress. However, anaerobic respiration is significantly less efficient in energy production, and the consumption of pyruvate increases sharply. Consequently, the rate of glycolysis was markedly enhanced, which may have contributed to compensating for energy deficits via increased carbohydrate consumption [37,38]. The waterlogging tolerance of ‘Shuixiu’ was reflected in its systemic metabolic reprogramming capability. Similar to reports of the glycolytic pathway being broadly activated in kiwifruit ‘Donghong’ and ‘Hayward’ after flooding [39,40], ‘Shuixiu’ also reinforced this pathway. However, this study further reveals that the regulation in ‘Shuixiu’ was more precise and coordinated: rather than simply upregulating the entire pathway, it specifically stabilizes or enhances the activity of multiple key nodes—ranging from substrate input (GPI) through flux control (PFK) to energy output (aldolase, PGK, PGAM, and PK). This strategy of ‘pathway-wide reinforcement’ likely enabled efficient and smooth conversion of carbon flux into energy and substrates required to maintain vital activities, which may have contributed to alleviating the energy deficit inherent to anaerobic respiration. In contrast, the down-regulation of genes at multiple nodes in ‘Hongyang’ may have led to metabolic bottlenecks, resulting in interrupted carbon flow and failure in energy supply. This efficient glycolytic flux not only directly supplied ATP but also ensured the provision of the core metabolite—pyruvate.

As the core of aerobic respiration, the tricarboxylic acid (TCA) cycle directly affects plant energy supply efficiency. Root hypoxia from waterlogging inhibited aerobic respiration, weakening stress resistance [41,42]. This study found 29 TCA-related genes with significant differential expression under waterlogging: most were downregulated in ‘Hongyang’ and ‘Shuixiu’, suggesting that hypoxia was associated with transcriptional repression of TCA-related genes, which might contribute to the adjustment of energy status under stress. Notably, core genes (encoding PDH E1 subunit, CS, and MDH) were specifically upregulated in SX, implying that ‘Shuixiu’ may alleviate the inhibition of aerobic respiration-related processes partly through maintaining the expression of key TCA genes. PDH, a key rate-limiting enzyme linking glycolysis and TCA, regulated pyruvate-to-acetyl-CoA conversion and energy yield [43]. ‘Shuixiu’ had higher basal PDH activity than ‘Hongyang’, with a 292% increase post-waterlogging and synchronized acetyl-CoA elevation. These results support the notion that ‘Shuixiu’ maintains a relatively higher aerobic respiration capacity, which may be linked to the activation of PDH under stress. Consistent with previous studies [37], maintaining PDH activity may reduce the accumulation of toxic anaerobic metabolites. Thus, the coordinated upregulation of PDH activity and acetyl-CoA level in ‘Shuixiu’ is likely associated with its better waterlogging tolerance, potentially via a more efficient aerobic energy supply.

Flooding-induced root hypoxia severely inhibits mitochondrial respiratory chain function, triggering a critical redirection of pyruvate metabolic flux. On one hand, pyruvate is channeled into fermentation pathways catalyzed by LDH or ADH, producing lactate or ethanol while regenerating NAD^+^. This process sustains glycolysis to partially compensate for the energy deficit [41,44]. Previous studies have shown that in *A. valvata* Dunn, two ERF transcription factors, *AcERF74/75*, specifically upregulate the expression of the ADH gene *AcADH1*, which is associated with enhanced ethanol fermentation efficiency and may contribute to waterlogging tolerance [39]. Compared to the less tolerant *A. chinensis*, the more waterlogging-tolerant *A. deliciosa* exhibited a more pronounced upregulation in the activities of PDC and ADH, as well as ethanol content in the roots. This further suggested that differences in low-oxygen tolerance among kiwifruit germplasm may be associated with variations in their ethanol fermentation metabolic potential [22]. In this study, the ADH-encoding genes in both ‘Shuixiu’ and ‘Hongyang’ were significantly upregulated under waterlogging stress, aligning with the aforementioned mechanism. However, compared to ‘Shuixiu’, ‘Hongyang’ showed upregulation in a greater number of ADH genes. Furthermore, ethanol content measurements revealed a rapid and substantial accumulation of ethanol in the roots of ‘Hongyang’ after flooding, whereas the ethanol content in SX roots exhibited an overall declining trend. This indicated a divergence in energy supply strategies under hypoxic stress between the two cultivars, with ‘Hongyang’ potentially relying more heavily on the ethanol fermentation pathway to sustain energy supply. Nevertheless, excessive ethanol accumulation could inflict multidimensional damage on plant roots, posing a potential risk that limited the enhancement of waterlogging tolerance. As a small lipophilic molecule, ethanol could penetrate the phospholipid bilayer of root cell membranes, disrupting membrane lipid structure and fluidity [45,46]. Concurrently, ethanol inhibited mitochondrial function and the activity of key metabolic enzymes, interfering with the coordinated operation of glycolysis and the respiratory chain [44]. Additionally, ethanol accumulation can indirectly affect carbon-nitrogen metabolic balance, competing for resources with core energy metabolism pathways, ultimately impairing root absorption and transport functions and negating the short-term adaptive advantages conferred by fermentation [47]. Given the superior waterlogging tolerance of ‘Shuixiu’ and the well-documented toxicity of ethanol, the distinct ethanol accumulation patterns between the two cultivars are of particular interest. The lower ethanol content in ‘Shuixiu’ roots likely alleviates the adverse effects associated with ethanol toxicity [48]. By avoiding excessive ethanol buildup, ‘Shuixiu’ may better preserve membrane integrity, maintain mitochondrial function, and sustain the coordinated operation of glycolysis and downstream metabolic pathways under hypoxic conditions. Such avoidance of ethanol-induced metabolic disruption could contribute to the superior waterlogging tolerance exhibited by ‘Shuixiu’ relative to ‘Hongyang’. However, the specific physiological and molecular mechanisms underlying the differential ethanol accumulation between the two cultivars remain to be elucidated.

The pyruvate generated by glycolysis could also undergo transamination catalyzed by transaminases such as glutamate-pyruvate transaminase (GPT) [49]. In this reaction, pyruvate reacts with glutamate to produce alanine and α-ketoglutarate. Although this pathway does not yield ATP, it effectively prevents the accumulation of toxic metabolites such as pyruvate, lactate, ethanol, and ammonia (which tends to accumulate due to disrupted nitrogen metabolism under hypoxia), thereby potentially alleviating ammonia-induced cellular damage [50]. Furthermore, upon reoxygenation, alanine could release pyruvate and glutamate through reverse transamination, re-entering carbon and nitrogen metabolism [51,52]. In the present study, the ‘Shuixiu’ significantly enhanced the alanine fermentation pathway through precise regulation of gene expression and enzyme activity in pyruvate metabolism. This regulatory mechanism provided two core advantages: First, compared with ‘Hongyang’, ‘Shuixiu’ directed more pyruvate toward alanine synthesis, substantially reducing the conversion of pyruvate to ethanol via ADH [49]. Consequently, ethanol accumulation in roots was markedly lower, which mitigated ethanol-induced damage—including permeabilization of the plasma membrane and inhibition of respiratory enzymes—at its source. Second, the substantial synthesis of alanine acted as a ‘temporary storage pool’ for pyruvate [53]. This not only prevented the feedback inhibition of glycolysis caused by excessive cytosolic pyruvate accumulation but also maintained a stable carbon metabolic flux, thereby supporting basic cellular functions under hypoxic conditions.

## 4. Materials and Methods

### 4.1. Plant Material and Stress Treatment

*Actinidia valvata* ‘Shuixiu’ was selected from wild individuals of *Actinidia valvata* collected in the wild by our team. It was identified as a waterlogging-tolerant elite genotype and preserved in the Kiwifruit Germplasm Repository of Jishou University. *Actinidia chinensis* ‘Hongyang’ was obtained from the Guangxi Institute of Botany. Seedlings with uniform growth, intact and healthy leaves (without etiolation, wilting, disease spots, or insect damage), well-developed root systems (without rot or deformity), and overall consistent vegetative growth with no significant differences were selected for pot cultivation. The nursery pots had a diameter of 20 cm. The culture substrate was prepared by mixing garden soil and vermiculite at a volume ratio of 2:1. No fertilizer was applied during cultivation, and seedlings were grown at room temperature. Artificial waterlogging treatment was performed. Potted seedlings were placed into another pot with a diameter of 22 cm. Water was added until the water level was 1 cm above the soil surface in the nursery pot, and this water level was maintained throughout the treatment. Seedlings were subjected to four waterlogging durations: 0 h, 6 h, 24 h, and 120 h. Fibrous root samples were collected at each time point. Three independent seedlings were used as biological replicates for each treatment, and 5 g of fibrous roots was collected from each plant. Samples were immediately frozen in liquid nitrogen and then stored at −80 °C until further analysis. ‘Hongyang’ seedlings were used as the control and subjected to exactly the same waterlogging treatments and sampling procedures as ‘Shuixiu’.

### 4.2. Determination of Physiological Indices Based on Spectrophotometry

The enzyme activities and metabolite contents were determined using spectrophotometric methods. All assays were performed strictly in accordance with the protocols provided with the commercial assay kits (Suzhou Keming Biotechnology Co., Ltd., Suzhou, China; www.cominbio.com (accessed on 2 March 2021), and Beijing Solarbio Science and Technology Co., Ltd., Beijing, China; www.solarbio.com (accessed on 2 March 2021)).

#### 4.2.1. Determination of Flooding-Related Enzyme Activities

The activities of hexokinase (HK), phosphoglycerate kinase (PGK), lactate dehydrogenase (LDH), alcohol dehydrogenase (ADH), alanine aminotransferase (ALT), and pyruvate dehydrogenase (PDH) were determined by monitoring the absorbance change in the coenzyme NAD(P)H at 340 nm. The activities of superoxide dismutase (SOD), catalase (CAT), and peroxidase (POD) were measured according to the corresponding kit protocols at wavelengths of 560 nm, 405 nm, and 470 nm, respectively.

#### 4.2.2. Determination of Flooding-Related Metabolite Contents

Ethanol and glucose levels were determined using colorimetric assay kits at 505 nm. Pyruvate content was measured at 520 nm. Starch content was quantified via the iodine-starch colorimetric method at 620 nm. The superoxide anion generation rate and malondialdehyde (MDA) content were detected according to the kit instructions at 530 nm and 532 nm, respectively.

### 4.3. LC-MS Analysis of Key Metabolites in Glycolysis, TCA Cycle, and Alanine Metabolism

The contents of glucose-6-phosphate, fructose-1,6-bisphosphate, 3-phosphoglycerate, alanine, malate, and oxaloacetate were determined using liquid chromatography-mass spectrometry (LC-MS). Samples were ground in liquid nitrogen, and 50–200 mg was accurately weighed and extracted with 1 mL of pre-cooled extraction solvent (methanol:acetonitrile:water = 2:2:1, *v*/*v*/*v*). The mixture was subjected to ice-bath ultrasonication for 30 min, repeated once, and then incubated at −20 °C for 1 h to precipitate proteins. After centrifugation at 12,000× *g* and 4 °C for 20 min, the supernatant was collected and filtered through a 0.22 μm microporous membrane. The filtrate was vacuum-dried, reconstituted in 200 μL of an acetonitrile-water solution (1:1, *v*/*v*), and centrifuged again at 12,000× *g* and 4 °C for 20 min. The final supernatant was injected for analysis.

Chromatographic separation was performed on a Thermo Vanquish ultra-performance liquid chromatography system equipped with a Waters (Milford, MA, USA) ACQUITY BEH Amide column (100 mm × 2.1 mm, 1.8 μm). The column temperature was maintained at 40 °C, with a flow rate of 0.3 mL/min and an injection volume of 2 μL. The mobile phase consisted of 10 mM ammonium acetate in water (A) and acetonitrile (B), using the following gradient elution program: 0–9 min, 90% to 40% B (linear); 9–9.1 min, 40% to 90% B (linear); 9.1–12 min, 90% B (held). Mass spectrometric detection was carried out using a Thermo Q Exactive high-resolution mass spectrometer (Waltham, MA, USA), with data acquired in electrospray ionization (ESI) negative mode.

### 4.4. RNA-Seq Analysis

Transcriptome sequencing and analysis were performed as previously described [54]. Briefly, the raw sequencing reads generated from the Illumina HiSeq platform (San Diego, CA, USA) were filtered using FASTP v.0.23.2 with the parameter settings as follows: n_base_limit 15, qualified_quality_phred 20 to remove adapter sequences, low-quality sequences, and short sequences. The obtained high-quality clean reads were aligned to the *A. chinensis* ‘Hongyang’ V1 reference genome [55] using HISAT2 v.2.2.2.1 with the default parameters. Novel gene prediction was conducted using StringTie v2.1.6, and the aligned read counts of genes were quantified using featureCounts v2.0.3 [54]. The FPKM values of each gene were then calculated based on the gene length. Differential expression analysis between the two sample groups was carried out using DESeq2 v1.22.1. The *p* values were adjusted by the Benjamini & Hochberg method, and the adjusted *p* value < 0.05 and |log2FoldChange| ≥ 1 were used as the thresholds for screening significantly differentially expressed genes.

### 4.5. Statistical Analyses

Statistical analyses were performed with SPSS 19.0 (SPSS Inc., Armonk, NY, USA). Two-tailed Student’s *t*-tests were applied to assess group differences. Data were presented as mean ± standard error (SE) from at least three independent replicates. Graphs were generated using GraphPad Prism 7 (GraphPad Software Inc., Boston, MA, USA).

## 5. Conclusions

This study clarified that the waterlogging tolerance mechanism of the kiwifruit cultivar ‘Shuixiu’ originates from the synergistic regulation of multiple core metabolic pathways. It specifically upregulated the expression of sucrose synthase and α/β-amylase genes to accelerate the degradation of sucrose and starch into hexoses, thereby generating glucose-6-phosphate (G6P), the initial substrate for glycolysis, to ensure the supply of energy metabolic substrates. Meanwhile, it may avoid ATP consumption and carbon source competition associated with excessive trehalose synthesis by inhibiting the sustained high expression of the TPS genes, which likely ensures that more glucose flows toward G6P. In the glycolytic pathway, ‘Shuixiu’ targeted enhancing the key node genes related to substrate input (GPI), flux control (PFK), and energy output (aldolase, PGK, PGAM, and PK), guaranteeing the efficient conversion of carbon flux into ATP and pyruvate. For pyruvate metabolism, it converts pyruvate into alanine for temporary storage by restricting ethanol production and enhancing the alanine fermentation pathway, thereby reducing the accumulation of toxic metabolites and maintaining metabolic homeostasis. In addition, under hypoxic conditions, ‘Shuixiu’ could maintain the expression of core tricarboxylic acid (TCA) cycle genes such as PDH E1 subunit and citrate synthase, which may help retain partial aerobic respiration capacity. The synergistic effect of these pathways established the waterlogging-tolerant metabolic network of ‘Shuixiu’. These findings elucidated the core metabolic pathways underlying kiwifruit waterlogging tolerance, providing molecular targets for the breeding of waterlogging-tolerant cultivars.

## Data Availability

The data presented in this study are available on request from the corresponding author. The data are not publicly available due to copyright restrictions.

## Supplementary Materials

The following supporting information can be downloaded at: https://www.mdpi.com/article/10.3390/ijms27073147/s1.
